# Cement injection and postoperative vertebral fractures during vertebroplasty

**DOI:** 10.1186/s13018-019-1273-z

**Published:** 2019-07-19

**Authors:** Le Hu, Hao Sun, Hua Wang, Jun Cai, Yuping Tao, Xinmin Feng, Yongxiang Wang

**Affiliations:** 1 Department of Orthopedics, Clinical Medical College of Yangzhou University, Yangzhou, 225001 China; 2 Department of Orthopedics, Northern Jiangsu People’s Hospital, Clinical Medical School, Yangzhou University, 98 West Nangtong Road, Yangzhou, 225001 Jiangsu China; 3 The Second Clinical College of Dalian Medical University, Dalian, 116000 China

**Keywords:** Osteoporotic vertebral compression fractures (OVCF), Percutaneous vertebroplasty, Bone cement injection rate, Adjacent vertebral fractures

## Abstract

**Objective:**

Vertebroplasty is the most widely used method for treating osteoporotic vertebral compression fractures (OVCF). During this procedure, bone cement is injected into the vertebral body. Fracture and additional fractures can occur adjacent to the treatment site. Thus, we studied factors causing such vertebral fractures after vertebroplasty and calculated the appropriate amount of bone cement to inject.

**Methods:**

From September 2012 to March 2016, 187 patients with OVCF undergoing vertebroplasty were selected, and 112 patients with complete follow-up information were selected. Of these, 28 had adjacent vertebral fractures (refracture group) during the follow-up period, and 84 patients had no adjacent vertebral fractures (control group). Then, sex, age, body weight, bone mineral density (BMD), and bone cement injection (bone cement injection volume and bone fracture vertebral volume percent) were compared.

**Results:**

All patients had significant pain relief within 24 h (preoperative and postoperative [24 h later] VAS scores were 7.4 ± 0.8 and 2.3 ± 0.5, respectively). The age and weight were not statistically significantly different (*P* > 0.05). BMD values were statistically significantly different between groups as was sex (*P* < 0.05).

**Conclusions:**

Bone cement injection volume, BMD values, and sex were statistically significantly related to adjacent vertebral fractures after vertebroplasty, and cement injection volumes exceeding 40.5% caused adjacent vertebral fractures.

## Introduction

As the population ages, osteoporotic vertebral compression fractures (OVCF) are increasing, as are acute and chronic pain episodes and progressive spinal deformities. It reduces the quality of life, impairs physical function, and increases mortality. Thus, the optimized treatment of OVCF is desired [1]. Vertebroplasty is an effective therapy for OVCF, and it reduces pain and prevents an additional collapse of the vertebral body via injection of bone cement into the fracture to fix the fractured vertebral body. However, some patients have immediate post-operative fractures of the adjacent vertebral body, and this is undesirable [2–5]. It is likely that the distribution of cement and the injection volume cause vertebral stiffness, which can cause refractures [6, 7].

Current data suggest that bone cement injection volume and pain relief were positively correlated, so cement volumes exceeding the vertebral body volume were used for 27.8% of treated patients [8]. However, we now know that excessive bone cement volumes can increase the risk of bone cement leakage and increase vertebral body stiffness, causing adjacent vertebral fractures [7, 9]. Therefore, the bone cement injection volume should be optimized.

## Materials and methods

From September 2012 to March 2016, 187 OVCF patients were offered vertebroplasty in the Department of Orthopedics at Jiangsu Subei People’s Hospital. Patients with more than two vertebral fractures and those lost to follow-up were excluded, and a retrospective analysis of 112 patients was performed. CT and MRI were performed preoperatively to confirm the diagnoses and ensure that there was no spinal canal compression. Before the vertebroplasty, VAS scores and BMD values were measured.

Vertebroplasty was performed under C arm fluoroscopy. During surgery, patients were monitored for ECG, blood pressure, pulse, and blood oxygen. Vertebroplasty was performed by four orthopedic physicians who used a standard approach for surgery, general anesthesia, a bilateral pedicle approach, transpedicular, or parapedicular trajectories were used in all cases. Eleven- or 13-gauge bone-biopsy needles were advanced into the central aspect of the vertebral bodies for unipediculate approaches, and polymethyl methacrylate cement infused from both sides of the pedicle. During surgery, efforts were made to ensure that no bone cement leakage occurred. If leakage occurred, the surgery was stopped. Then, 24 h after surgery, patients were asked to walk with a stent. Routine X-ray examination was used to view the spine if there were any cement leaks after vertebroplasty. All operations were performed by the same operator, and all patients were treated with Mendec Spine Resin cement. All patients were followed up for 6 to 12 months, with an average of 9.1 months. X-ray examination of the vertebral body was performed in 1, 3, 6, and 12 months. If the patients had back pain after an operation, they were immediately consulted in the clinic and underwent the X-ray examination.

Calculation of the fractured vertebral body and bone cement volumes is as follows: Digital Imaging and Communications in Medicine (DICOM) images of thin-layer CT were calculated using Mimic 14.1 software (Materialize Software, Belgium), and bone cement and vertebral body volumes were calculated using a 3D calculation function.

### Statistical analysis

Statistical analysis was performed using SPSS 22 software (vendor missing as is location). Age, weight, and BMD values were analyzed using an independent Student *t* test, and gender was analyzed using a Chi-squared test. Bone cement injection volume, a ROC curve, and risk factors between groups were analyzed.

## Results

Bone cement injection was successful for all patients (Table 1), and no cardiovascular or cerebrovascular events occurred during the perioperative period. All patients had significant pain relief within 24 h (preoperative and postoperative [24 h later] VAS scores were 7.4 ± 0.8 and 2.3 ± 0.5, respectively).

**Table 1:** Patients and vertebrae treated

Vertebral level treated	*n*
T7	3
T8	1
T9	5
T10	7
T11	15
T12	21
L1	26
L2	19
L3	7
L4	6
L5	2
Total	112

**Fig. 1:**
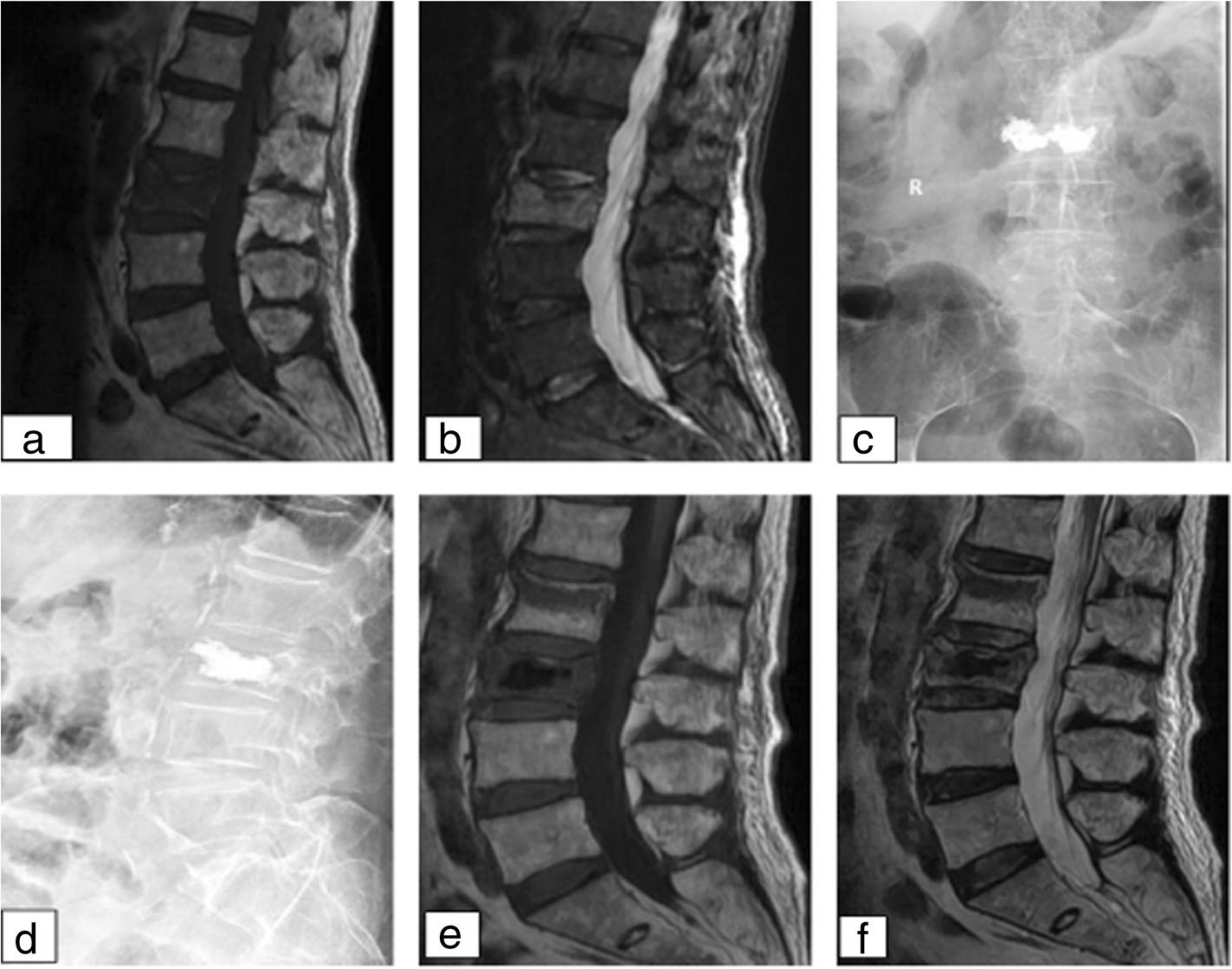
Imaging data of fractures after 1 month of PVP

Patients were divided into refracture (adjacent vertebral fracture during follow-up) and control groups (no adjacent vertebral fracture during follow-up). The first fracture in refracture group was 1 month after surgery (Fig. 1), with an average of 5.9 months. Follow-up results showed that 28 had adjacent vertebral fractures (refracture group) during the follow-up period, and 84 patients had no adjacent vertebral fractures (control group). Among them, there were 12 cases of operative upper vertebral refracture and 16 cases of operative lower vertebral refracture. The earliest occurrence of adjacent vertebral fracture was 1 month after the operation, with an average of 5.9 months.

**Fig. 2:**
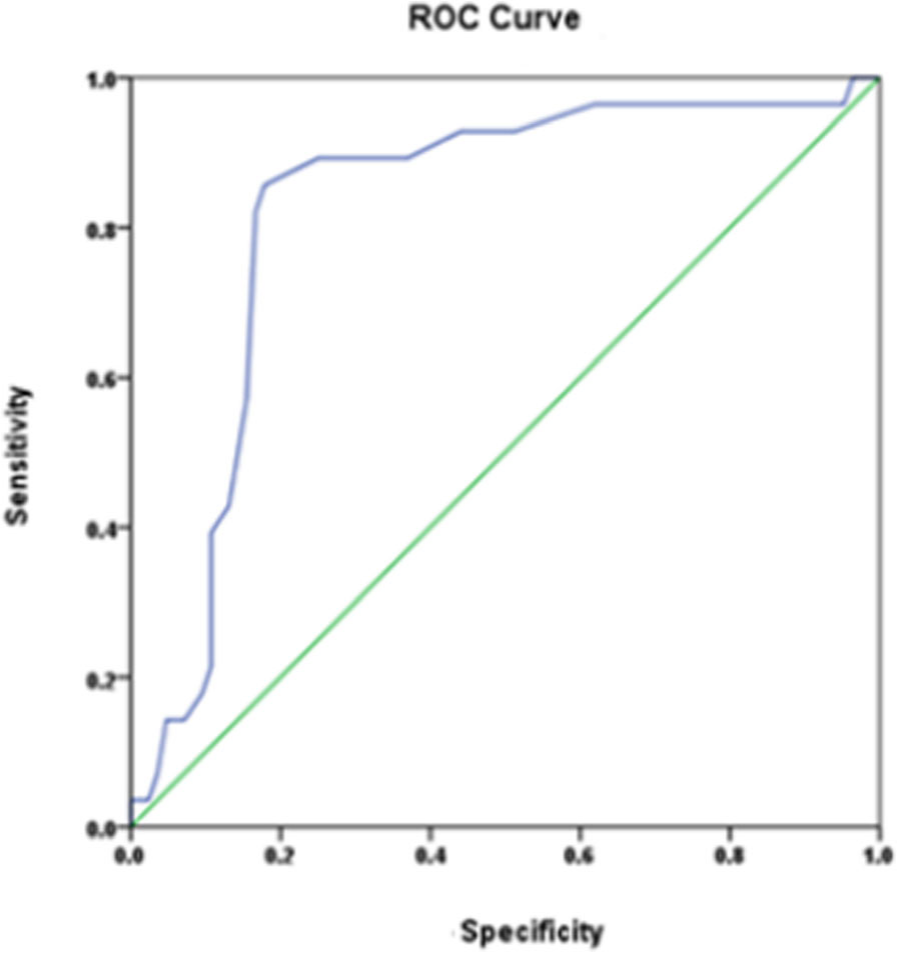
ROC curve of bone cement injection rate

SPSS 22 software was used to analyze groups, and age and weight were not statistically significantly different. BMD values were statistically significantly different between groups as was sex (Table 2). Sex may be tied to osteoporosis, which is another variable to consider. Bone cement volume was analyzed (Fig. 2, Table 3).

**Table 2:** Comparison of groups

Groups	*n*	Sex		Age (*x* ± *s*)	BMD	Weight
		Male	Female			
Controls	84	35	49	73.15 ± 7.94	−2.63 ± 0.3	53.16 ± 4.35
Refractures	28	5	23	75.75 ± 7.56	−2.8 ± 0.26	51.43 ± 4.85
*χ*^*2*^/*t*	–	5.185		1.516	2.681	1.767
*p* value	–	0.023		0.132	0.008	0.080

**Table 3:** Bone cement infusion and fractures of adjacent vertebral body after vertebroplasty

Cut-off value	AUC	*p*	Sensitivity (%)	Specificity (%)	Youden index
0.405	0.823	< 0.001	85.7	82.1	0.678

## Discussion

We analyzed recurrent adjacent vertebral body fractures in patients with OVCF after vertebroplasty, and bone cement volume, BMD values, and sex were correlated with postsurgical refracture. Vertebroplasty is commonly used to treat OVCF because it is simple, offers little trauma, and affords pain relief [10, 11]. Multiple studies of adjacent vertebral fractures in patients undergoing vertebroplasty indicate that bone cement volume contributes to refracture risk [7, 12–14]. Kwon’s group reported [8] that as much bone cement as possible should be used to treat OVCF, and the volume of the vertebral body exceeds 27.8% of the transfusion volume, recovery is optimal. Other studies [15, 16] suggest that bone cement volume is positively related to postoperative pain relief, and they also recommend the greatest volume of cement. These studies do not consider the long-term consequences and subsequent adjacent vertebral fractures. Excessive injection of bone cement increases the stiffness of the vertebral body, which can cause fracture and increase the risk of bone cement leakage [7, 17].

Zhu’s group [9] showed that to avoid leakage of cement, bone cement volumes for the thoracic spine should be < 3.5 ml, and for the lumbar spine, they should not exceed 4 ml. This ignores individual patient differences, vertebral body volume, and vertebral compression degrees, which are important for volume considerations. Many studies indicate that disc pressure leads to bone cement intradiscal leakage and may lead to a deflection of the adjacent vertebral endplate, resulting in fractures [2, 18, 19]. Using 3D finite element analysis of the spine, Kim’s group reported [20] that when bone cement volume reaches 30% of the volume of the vertebral body, the cement restores bone hardness; however, when the cement exceeds this volume, abnormal hardness results, and this increases spinal stress. Belkoff and colleagues suggest [21] that bone cement volumes of 2 ml can restore vertebral body stiffness. Vertebroplasty with bone cement not only increases vertebral strength but can also increase the stress of the adjacent vertebral body [22]. Currently, there is no bone cement volume standards that account for pain relief, additional fractures, injection volumes at different spinal locations, and bone strength restoration or vertebral compression [8, 9, 20, 21]. Thus, we used ROC curve analysis to predict optimal bone cement volume to prevent postoperative fractures and additional surgeries.

We noted that adjacent vertebral fractures after vertebroplasty were associated with low BMD and being female. Ryu’s group reported [3] that vertebroplasty alters spine biomechanics and increases pressure on the adjacent vertebral body, especially for women with severe osteoporosis. Many studies indicate that post-vertebroplasty fractures for postoperative patients with average BMD values are lower and that BMD is a risk factor for postoperative secondary adjacent vertebral fractures [4, 23, 24]. This is consistent with our research [4]. Also, studies show that adjacent vertebral fractures after vertebroplasty may be related to age and weight [2, 5], but we did not find this in our work.

Our study was limited by small sample size, and we did not analyze the location of the fractured vertebral body and the fracture type; both may affect the degrees of thoracolumbar motion and fracture. Vertebral fractures at multiple sites were not included, and we did not consider the extent of the fracture of the vertebral body or the bone cement distribution. Therefore, more work is required to optimize our preliminary findings.

## Conclusions

Bone cement injection volume, BMD values, and sex were significantly related to adjacent vertebral fractures after vertebroplasty. We also found that more women suffered from adjacent vertebral fractures and had lower BMD values. However, cement injection volume exceeding 40.5% caused adjacent vertebral fractures. Therefore, we suggest that the amount of bone cement should be calculated in advance according to the imaging data before the operation, and the amount of bone cement injected should be as much as possible but should not exceed 40.5%. It can shorten the operation time and should be actively treated with anti-osteoporosis therapy after the operation, especially for female patients. This can effectively alleviate back pain and reduce the risk of refracture of the adjacent vertebral body after surgery.

## Data Availability

Please contact the corresponding author for data requests.

## Abbreviations

BMD: Bone mineral density
OVCF: Osteoporotic vertebral compression fractures
